# Deglycosylated EpCAM regulates proliferation by enhancing autophagy of breast cancer cells via PI3K/Akt/mTOR pathway

**DOI:** 10.18632/aging.203795

**Published:** 2022-01-04

**Authors:** Liu Yang, Qijun Wang, Qian Zhao, Fan Yang, Tingjiao Liu, Xiaohua Huang, Qiu Yan, Xuesong Yang

**Affiliations:** 1 Department of Biochemistry and Molecular Biology, College of Basic Medicine Sciences, Dalian Medical University, Dalian, China; 2 Section of Oral Pathology, College of Stomatology, Dalian Medical University, Dalian, China; 3 Department of Clinical Biochemistry, College of Laboratory Science, Dalian Medical University, Dalian, China

**Keywords:** EpCAM, glycosylation, autophagy, PI3K/Akt/mTOR, breast cancer

## Abstract

Autophagy is an important regulator of cellular homeostasis and its dysregulation often results in cancer. Aberrant glycosylation induced by oncogenic transformation contributes to tumor invasion and metastasis. In a previous study, we have demonstrated that EpCAM, a glycosylation protein, is associated with cell growth and metastasis in breast cancer. But the effect of EpCAM glycosylation on autophagy is not clear. the precise mechanism of regulation remains largely unknown. In this study, breast cancer cells were transfected with N-glycosylation mutation EpCAM plasmid to express deglycosylated EpCAM. The result showed that deglycosylated EpCAM promoted autophagy in breast cancer cells. We further confirmed this conclusion with the activator (Rapamycin, RAP) and inhibitor (Wortmannin) of autophagy. We also found that deglycosylated EpCAM promoted apoptosis and inhibited proliferation through activating autophagy by suppressing Akt/mTOR signaling pathway in breast cancer cells. These findings represent a novel mechanism by which deglycosylated EpCAM inhibits proliferation by enhancing autophagy of breast cancer cells via PI3K/Akt/mTOR pathway. In conclusion, the combination of autophagy modulation and EpCAM targeted therapy is a promising therapeutic strategy in the treatment of breast cancer.

## INTRODUCTION

Breast cancer (BC) is the second most common malignancy among women [1]. The main cause of high mortality rate in BC is cancer recurrence, which origin from the metastasis of dormant tumor cells [2, 3]. Till now, surgical operation is the main treatment for breast cancer, which is effective for early stage of BC. But for the advanced, incurable stage of BC, transitional chemotherapeutic agents do not produce good results [4, 5]. Therefore, it is necessary to develop new targeted drugs for advanced BC as early as possible.

Epithelial cell adhesion molecule (EpCAM) is a glycoprotein expressing on the surface of epithelial cell and several tumor types, including colorectal cancer [6], endometrial carcinoma [7], lung carcinoma [8], gastric cancer, and BC [9]. It has been shown that there exists a close relationship between overexpression of EpCAM and advanced stages of BC [10]. Our study has demonstrated that decreased EpCAM caused a notable negative effect on cell proliferation, migration and invasion *in vitro* [11, 12]. EpCAM knockdown promoted apoptosis and raised the cytotoxic effect of 5-Fluorouracil in breast cancer cells through MAPK signaling pathway [13]. These results suggested that EpCAM has a close relationship with malignant biological behaviors. But up to now, the relationship between EpCAM and autophagy has not been clear.

Autophagy is a self-degradative process and is important to maintain cellular homeostasis, development, differentiation, cell survival and death, which has been found to play an interesting role in cancer biology [14]. Autophagy has two mutually contradictory roles in tumors. These dual effects mean that we could find autophagy upregulation as well as autophagy downregulation in cancers. Therefore, autophagy show dual properties during malignant transformation, including oncogenic and tumor suppressor properties [15, 16]. Thus, it is essential to identify the key autophagy targets for new therapeutic agents.

Glycosylation modification on protein is the popular common form of post-translational modification. Tumor cells are usually accompanied by glycosylated modifications, which result in inhibition of apoptosis, uncontrolled proliferation, and metastasis. Recent data suggest that through the process of autophagy glycoconjugates could regulate physiology. For example, serum proteins showed hypoglycosylation and autophagy downregulated when the X-linked ATP6AP2 was mutated in the mouse liver [17]. Autophagy is inhibited by excessive O-GlcNAcylation and harmful to neurons [18]. We have learned that EpCAM is a glycoprotein which has three glycosylation sites [19]. EpCAM showed different glycosylation in normal tissues and in head and neck tumors [20]. Based on the above, we inferred that glycosylation of EpCAM may play a role on autophagy in breast cancer. But the regulatory mechanism of EpCAM glycosylation on autophagy remains unclear. Therefore, whether glycosylated EpCAM is associated with proliferation and apoptosis caused by autophagy needs to be studied further.

Thus, the role and mechanism of deglycosylation EpCAM on autophagy will be discussed in this paper. Overall, our study supposes that targeting autophagy may become an effective treatment for breast cancer.

## RESULTS

### Effect of glycosylation of EpCAM on autophagy

**Figure 1:**
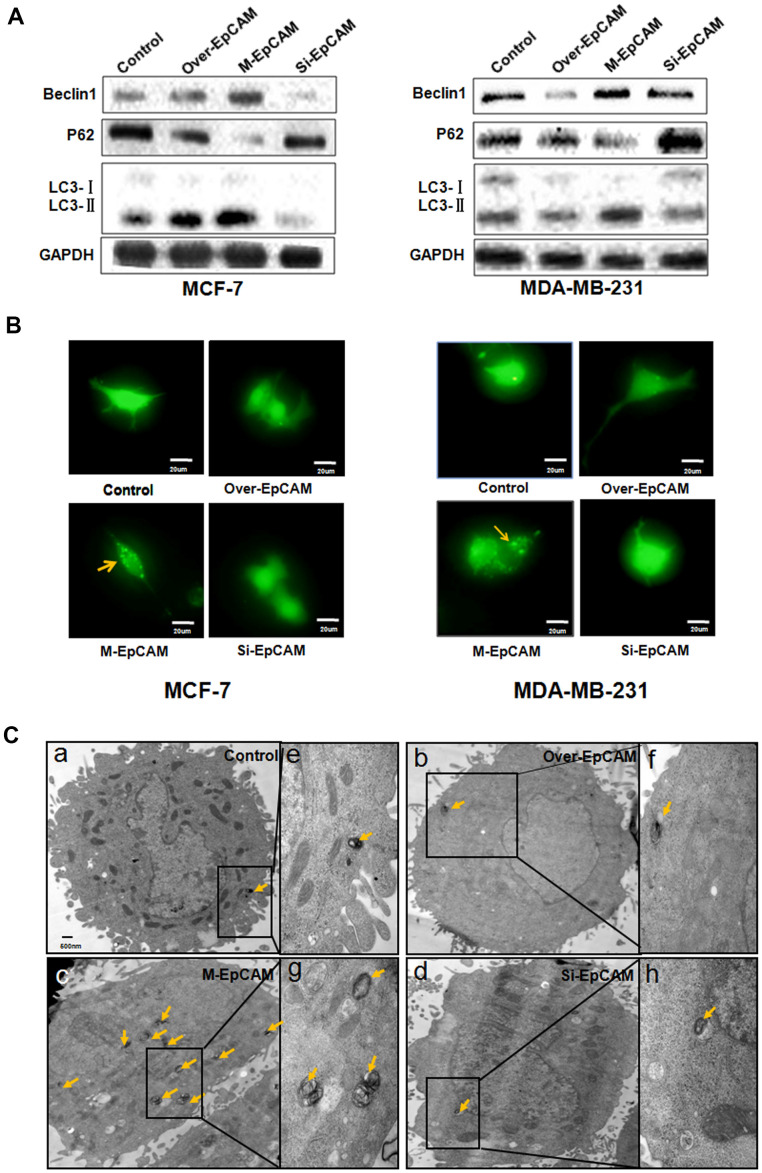
**Effect of EpCAM on autophagy.** (**A**) MCF-7 cells and MDA-MB-231 cells were treated with pCMV-SPORT 6-EpCAM plasmid, si-EpCAM sequence and M-EpCAM plasmid for 48 hr. Whole cell lysates were subjected to western blot to detect the expression of Beclin 1, p62 and the conversion from LC3-I to LC3-II. (**B**) MCF-7 cells and MDA-MB-231 cells were transfected with pCMV-SPORT 6-EpCAM plasmid or si-EpCAM sequence or M-EpCAM plasmid and pGFP-LC3 plasmid for 48 hr, images were collected. After transfection for 24 hr, the cells were observed under an inverted microscope. Arrow depicted the autophagosome. (**C**) Representative transmission electron microscopy images depicting ultrastructures of MCF-7 cells which were transfected with pCMV-SPORT 6-EpCAM plasmid, si-EpCAM sequence and M-EpCAM plasmid, respectively. (**e**–**h**) depicted boxed sections in panels (**a**–**d**) at a higher magnification, respectively. Arrows indicate autolysosomes.

To elucidate glycosylation of EpCAM in BC on autophagy, an EpCAM overexpression plasmid and small interfering RNA-mediated silencing of EpCAM (si-EpCAM) were used to increase and reduce EpCAM expression in MCF-7 and MDA-MB-231 breast cancer cell lines, respectively. N-glycosylation mutation of EpCAM plasmid was utilized to express deglycosylation EpCAM. Typical autophagosome markers, Beclin 1, P62 and LC3, were detected by using Western blot. As shown in Figure 1A, the expression of LC3 I and P62 reduced and the expression of LC3-II and Beclin 1 increased after treated with deglycosylation of EpCAM in these two cells (MCF-7 and MDA-MB-231), suggesting that glycosylation modification in EpCAM may be associated with autophagy.

In addition, we used a GFP-LC3 expression vector to analyze the autophagy activity of cells. The punctate green fluorescent proteins expressed by this vector were mainly concentrated on autophagic vacuoles. We detected autophagosomes by studying GFP-LC3 fluorescence with fluorescence microscope. The group transfected with M-EpCAM plasmid showed higher percentage of punctate GFP, while the groups transfected EpCAM overexpression plasmid, si-EpCAM sequence, with showed primarily diffused (Figure 1B). The results were consistent with those obtained for LC3-II levels in the western blot experiments. We inferred that N-glycosylation of EpCAM influenced the sub-cellular distribution of LC3.

When autophagy occurs, autophagosomes with double-membrane vesicles will be formed. The autophagosomes can swallow other organelles and send them to the lysosome. This process can be observed by transmission electron microscopy [21]. As shown in Figure 1C–1b, 1d, in the MCF-7 cells treated with EpCAM overexpression plasmid and si-EpCAM sequence, the cytoplasm was filled with organelles of high electron density, which were not contained within vacuoles. These results suggested that there should be short of organelle autophagy. In contrast, cytoplasmic vacuoles containing high electron density organelles were abundant around the nuclear in MCF-7 cells transfected with M-EpCAM plasmid. This result (Figure 1C–1c) supported the hypothesis that formation of autophagosome was regulated by glycosylation modification of EpCAM. Taken together, it demonstrated that in breast cancer cells autophagy is associated with glycosylation of EpCAM.

### Effect of regulators of autophagy on glycosylation of EpCAM

**Figure 2:**
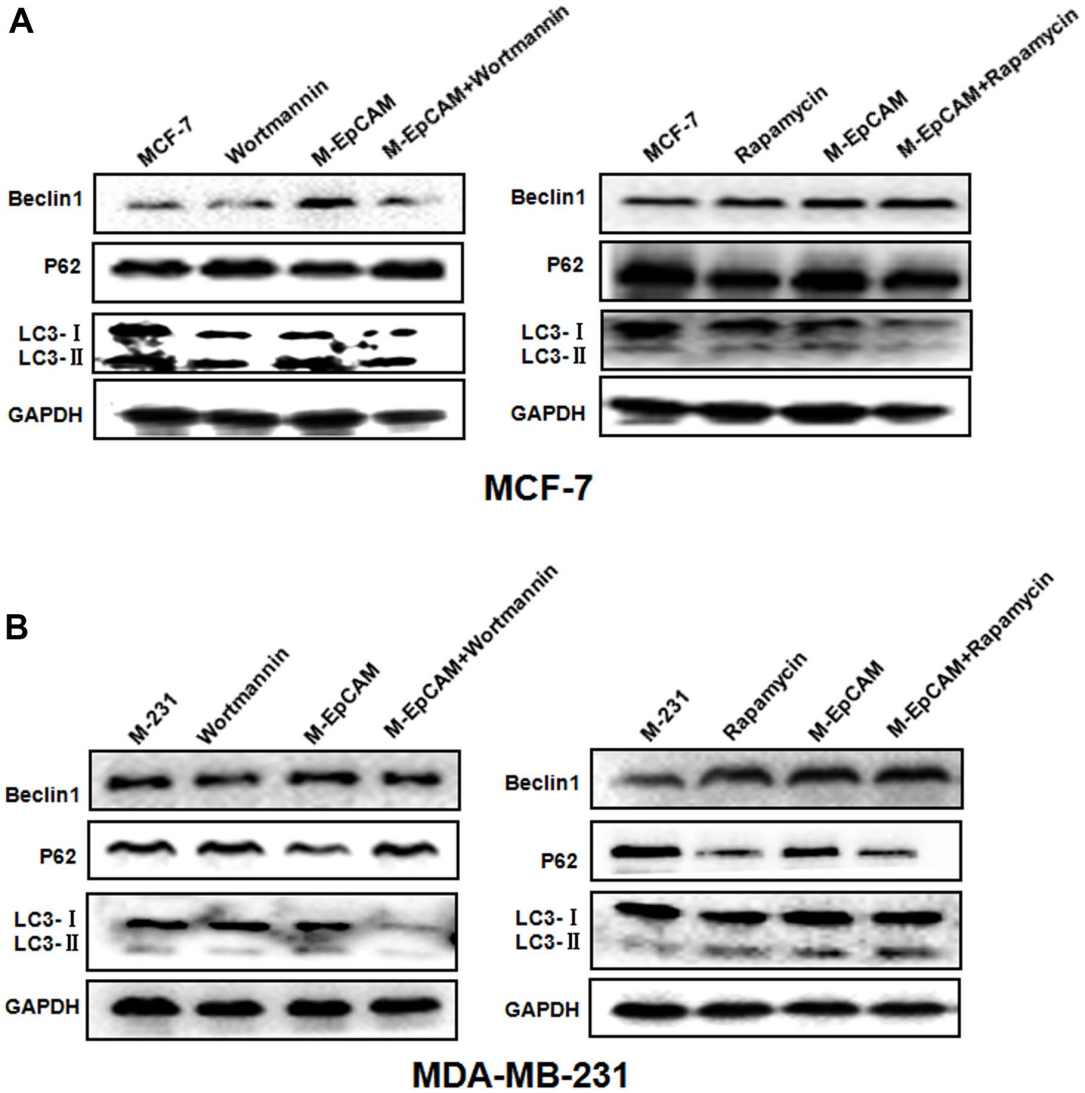
**Effect of regulators of autophagy on glycosylation of EpCAM breast cancer cells.** Treatment of MCF-7 (**A**) and MDA-MB-231 (**B**) cells were treated with 100 nM Wortmannin or 200 nM Rapamycin for 12 hr accompanied with transfected with M-EpCAM plasmid. Expressions of autophagy markers Beclin1, LC3, and p62 proteins were determined by western blot analysis.

Based on above results, we have inferred N-glycosylation of EpCAM is important for autophagy. Next, inhibitor (Wortmannin) and agonist (Rapamycin) of autophagy were used to confirm the conclusion fatherly. Cells were treated with 100 nM Wortmannin or 200 nM Rapamycin for 12 hr accompanied with transfected with M-EpCAM plasmid. These data (Figure 2) showed that the expression of Beclin1 and LC3-II were significantly decreased with Wortmannin treatment and increased with Rapamycin treatment for 12 hr, while the level of p62 had the opposite result. In addition, when Wortmannin accompanied with transfected with M-EpCAM plasmid, we found that the expressions of Beclin1 and LC3-II were decreased further and p62 was increased compared with treatment of M--EpCAM plasmid. We also found that Rapamycin accompanied with transfected with M-EpCAM plasmid, the expression of Beclin1 and LC3-II were increased further and p62 was decreased compared with treatment of M-EpCAM plasmid. Collectively, these results suggest that deglycosylated EpCAM regulated autophagy in breast cancer cells.

### The effect of inhibitor and activator of autophagy and deglycosylated EpCAM on apoptosis

**Figure 3:**
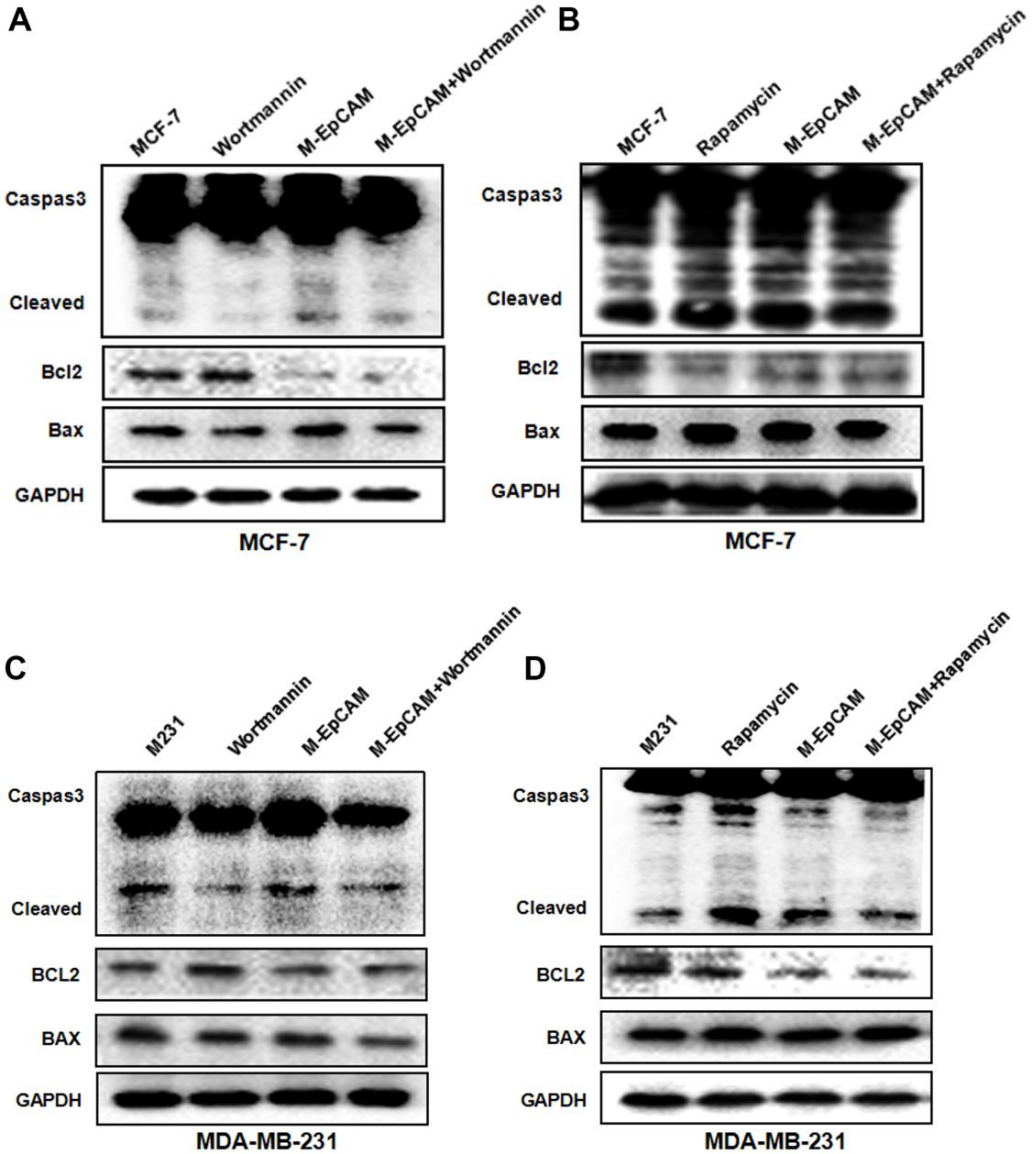
**Effect of regulators of autophagy and deglycosylated EpCAM on apoptosis in breast cancer cells.** (**A**, **B**) MCF-7 cells were incubated with 100 nM Wortmannin 200 nM Rapamycin for 12 hr after transfected with M-EpCAM plasmid. Expression of apoptosis related proteins Caspase 3, Bcl2 and Bax were detected with the method of Western blot. (**C**, **D**) MDA-MB-231 cells were incubated with 100 nM Wortmannin 200 nM Rapamycin for 12 hr after transfected with M-EpCAM plasmid. Expression of apoptosis related proteins Caspase 3, Bcl2 and Bax were detected with the method of Western blot.

We have demonstrated that deglycosylated-EpCAM strengthened the cytotoxic effect of 5-FU and promoted apoptosis in breast cancer cells [22]. It has been reported that autophagy is known to regulate cell cycle progression, survival and apoptosis [23]. Thus, we were interested in discussing the role of autophagy in deglycosylated EpCAM-mediated apoptosis in breast cancer cells. We used Wortmannin and Rapamycin to inhibit and activate autophagy, respectively. The result showed that apoptosis-related proteins cleaved-caspase 3 and Bax increased and anti-apoptotic protein Bcl2 decreased when cells were transfected with plasmid of M-EpCAM. After M-EpCAM transfected cells were incubated with 100 nM Wortmannin for 24 hr, the expression of cleaved-caspase 3 and Bax decreased and Bcl2 increased compared with the cells transfected with M-EpCAM plasmid only (Figure 3A, 3C). Next, we tested the effect of autophagy activator on the cell apoptosis. The western blot results showed that Rapamycin (activator) has the opposite effect compared to Wortmannin (inhibitor) (Figure 3B, 3D). Taken together, our findings proved that glycosylated EpCAM might regulate the apoptosis by influencing autophagy in breast cancer cells.

### The effect of inhibitor and activator of autophagy and deglycosylated EpCAM on proliferation

**Figure 4:**
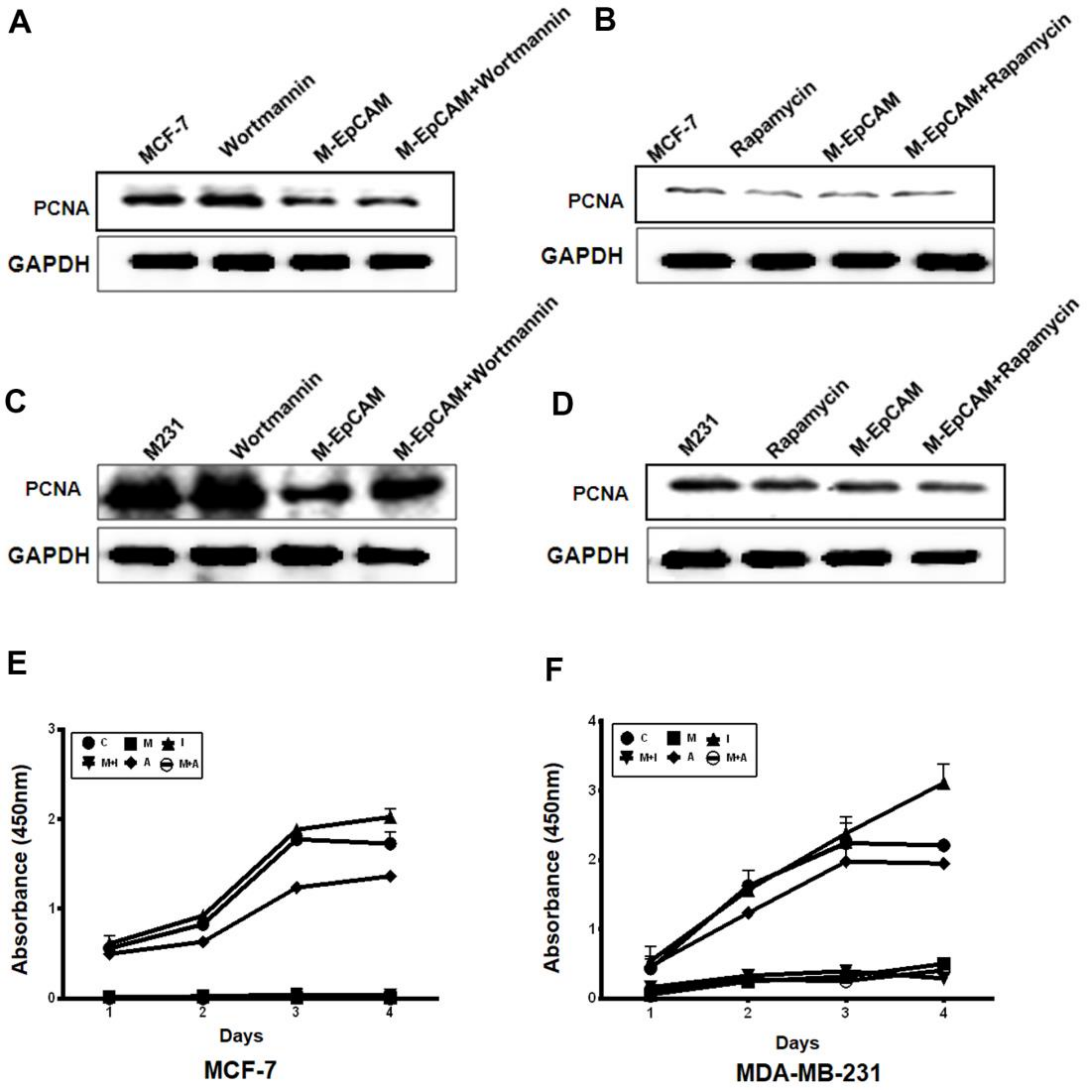
**Effect of regulators of autophagy and deglycosylated EpCAM on proliferation in breast cancer cells.** (**A**, **B**) MCF-7 cells were incubated with 100 nM Wortmannin or 200 nM Rapamycin for 12 hr after transfected with M-EpCAM plasmid. Expression of PCNA was detected with the method of Western blot. (**C**, **D**) MDA-MB-231 cells were incubated with 100 nM Wortmannin or 200 nM Rapamycin for 12 hr after transfected with M-EpCAM plasmid. Expression of PCNA was detected with the method of Western blot. (**E**, **F**) MCF-7 and MDA-MB-231 cells were incubated with 100 nM Wortmannin or 200 nM Rapamycin for 12 hr after transfected with M-EpCAM plasmid. The cells were cultured for another 4 days. The CCK8 assay used to evaluate the proliferation of the cells after transfection with the M-EpCAM plasmid or autophagic regulator.

Next, to further confirm that the effect of glycosylated of EpCAM may participate in proliferation through autophagy, we incubated MCF-7 and MDA-MB-231 cells with the autophagic inhibitor Wortmannin and stimulus Rapamycin, respectively. By monitoring the expression of proliferation maker PCNA, we found both cells showed Wortmannin increased and Rapamycin decreased the expression of PCNA. Deglycosylated EpCAM also decreased the PCNA expression. Synergistic effect of Wortmannin and deglycosylated EpCAM showed the callback of PCNA expression compared with using the Wortmannin only (Figure 4A, 4C). Then, we used Rapamycin (autophagic activator) to incubate these two breast cancer cells for 24 hr. The results showed that proliferation properties were inhibited after using the Rapamycin only. The results showed that proliferation properties were inhibited after using the Rapamycin only. Synergistic effect of Rapamycin and deglycosylated EpCAM showed the decrease of PCNA expression (Figure 4B, 4D). We also used CCK8 assay to evaluate the effect of glycosylated EpCAM and autophagy on proliferation, CCK8 assay results showed consistent with above western blot results of PCNA (Figure 4E, 4F). Over all, the finding suggested that glycosylated EpCAM might inhibit the proliferation through influencing autophagy in breast cancer cells.

### Deglycosylated EpCAM regulates autophagy, apoptosis and proliferation by PI3K/AKT/mTOR signaling pathway

**Figure 5:**
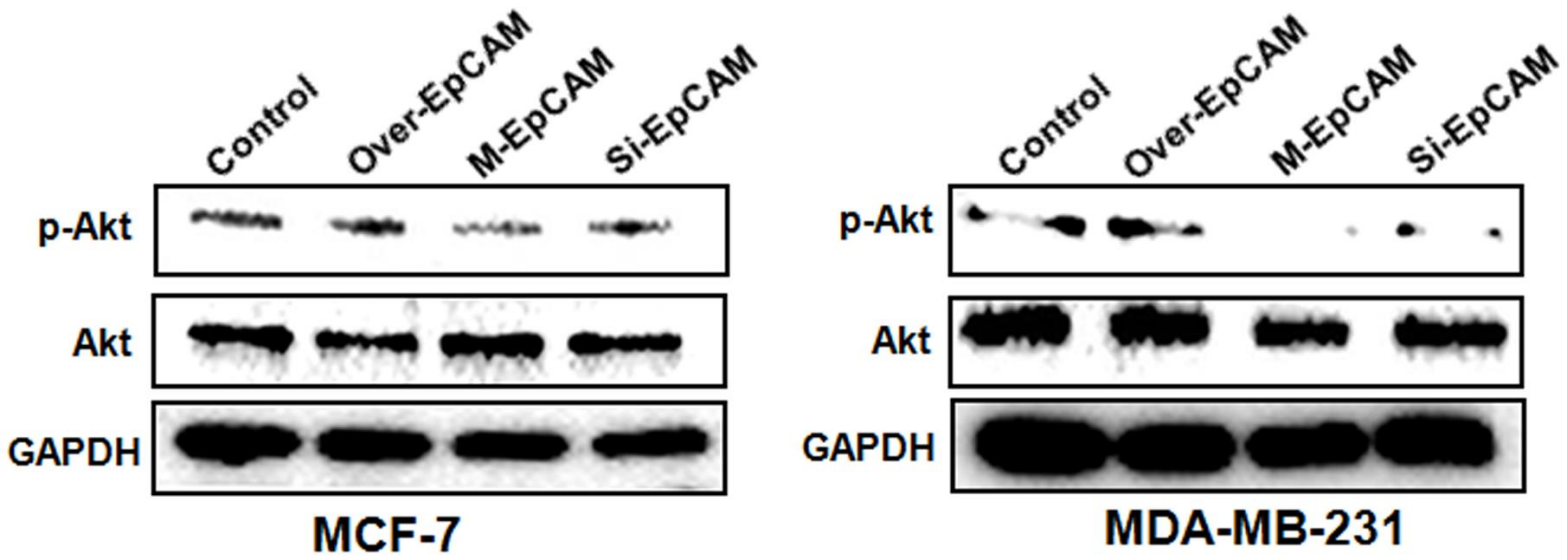
**Effects of EpCAM on the PI3K/AKT/mTOR signaling pathway in MCF-7 and MDA-MB-231 cells.** MCF-7 and MDA-MB-231 cells were transfected with pCMV-SPORT 6-EpCAM plasmid, si-EpCAM sequence and M-EpCAM plasmid for 48 hr, respectively. Expressions of pAkt and Akt were detected with method of Western blot.

**Figure 6:**
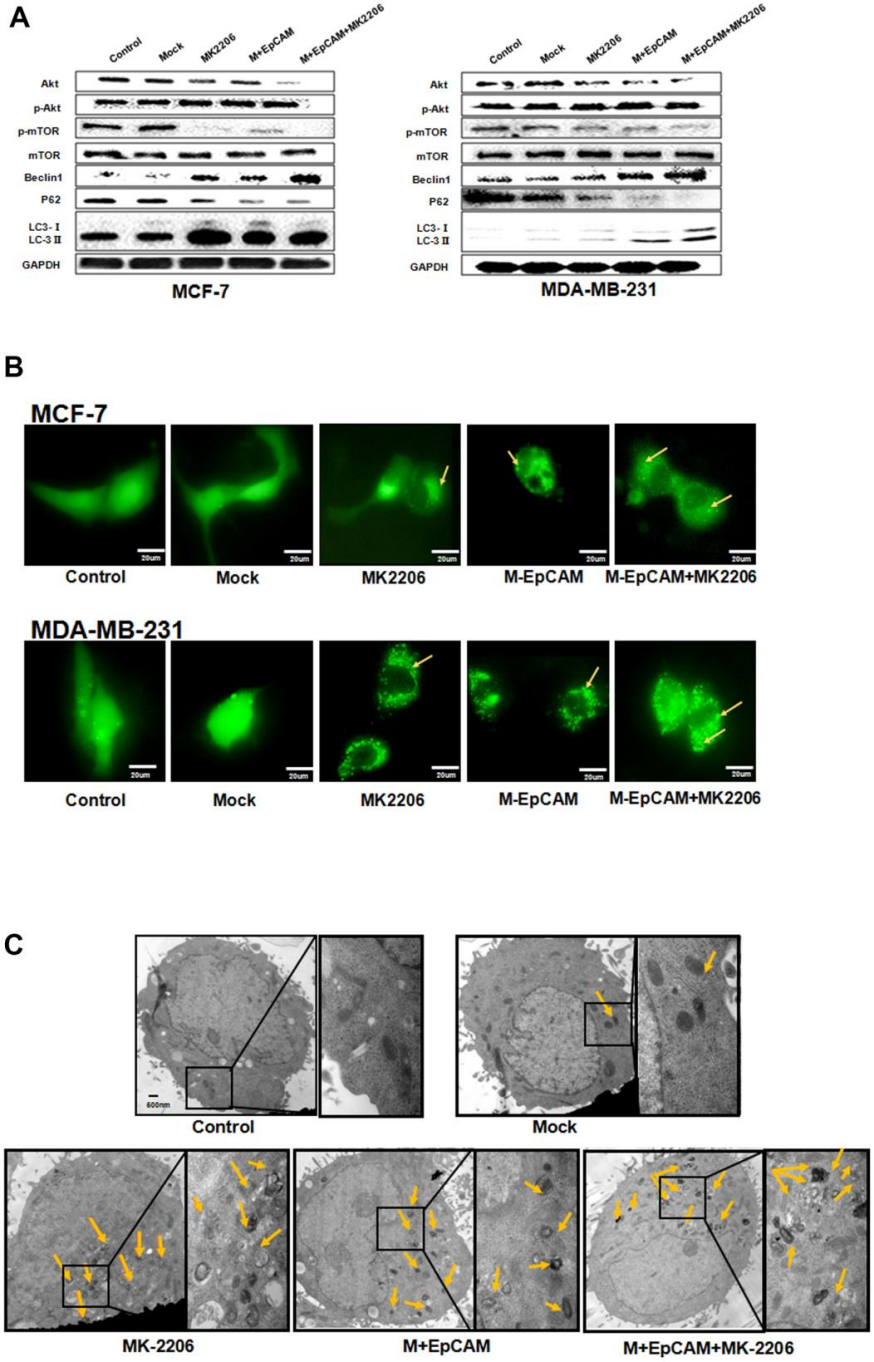
**Effects of EpCAM on autophagy via PI3K/AKT/mTOR signaling pathway in MCF-7 and MDA-MB-231 cells.** (**A**) MCF-7 cells and MDA-MB-231 cells were treated with M-EpCAM plasmid and MK2206 (1μM) for 48 h. Whole cell lysates were subjected to western blot to detect the expression of Beclin 1, p62 and the conversion from LC3-I to LC3-II. (**B**) MCF-7 cells and MDA-MB-231 cells were treated with M-EpCAM plasmid, pGFP-LC3 plasmid and MK2206 (1μM) for 48 hr. Cells were observed under an inverted microscope. Arrow depicted the autophagosome. (**C**) Representative transmission electron microscopy images depicting ultrastructures of MCF-7 cells which were transfected with M-EpCAM plasmid and MK2206 (1μM) for 48 h.

Many reports supported that autophagy is followed by the induction of apoptosis via PI3K/Akt/mTOR pathway [18, 24]. mTOR can be phosphorylated by phosphorylated-Akt to form p-mTOR, which plays a negative role in autophagy. Based on this, we explored the effect of glycosylation modification of EpCAM on Akt/mTOR signaling pathways. The result showed that deglycosylated EpCAM decreased the expression of pAkt (Figure 5). Next, to further confirm that PI3K/Akt/mTOR participated in this process, we incubated the cells with the Akt inhibitor MK2206 (1uM). The results showed that the levels of p-Akt and p-mTOR significantly declined when treated with MK2206. We also analyzed the effect of glycosylated EpCAM combined with MK2206. The results showed that deglycosylated EpCAM along with MK2206 decreased the expression of pAkt and pmTOR. Autophagy makers Beclin 1 and LC3-II increased and P62 and LC3-I decreased when treated with M-EpCAM plasmid and MK2206 (Figure 6A). Furthermore, fluorescence result showed that increased granulation of GFP-LC3 during synergistic effect between deglycosylation of EpCAM and Akt inhibitor MK2206 (Figure 6B). Transmission electron microscopy has the same results as above (Figure 6C). Taken together, we deduced that deglycosylated EpCAM promote autophagy via PI3K/AKT/mTOR signaling pathway in breast cancer cells.

**Figure 7:**
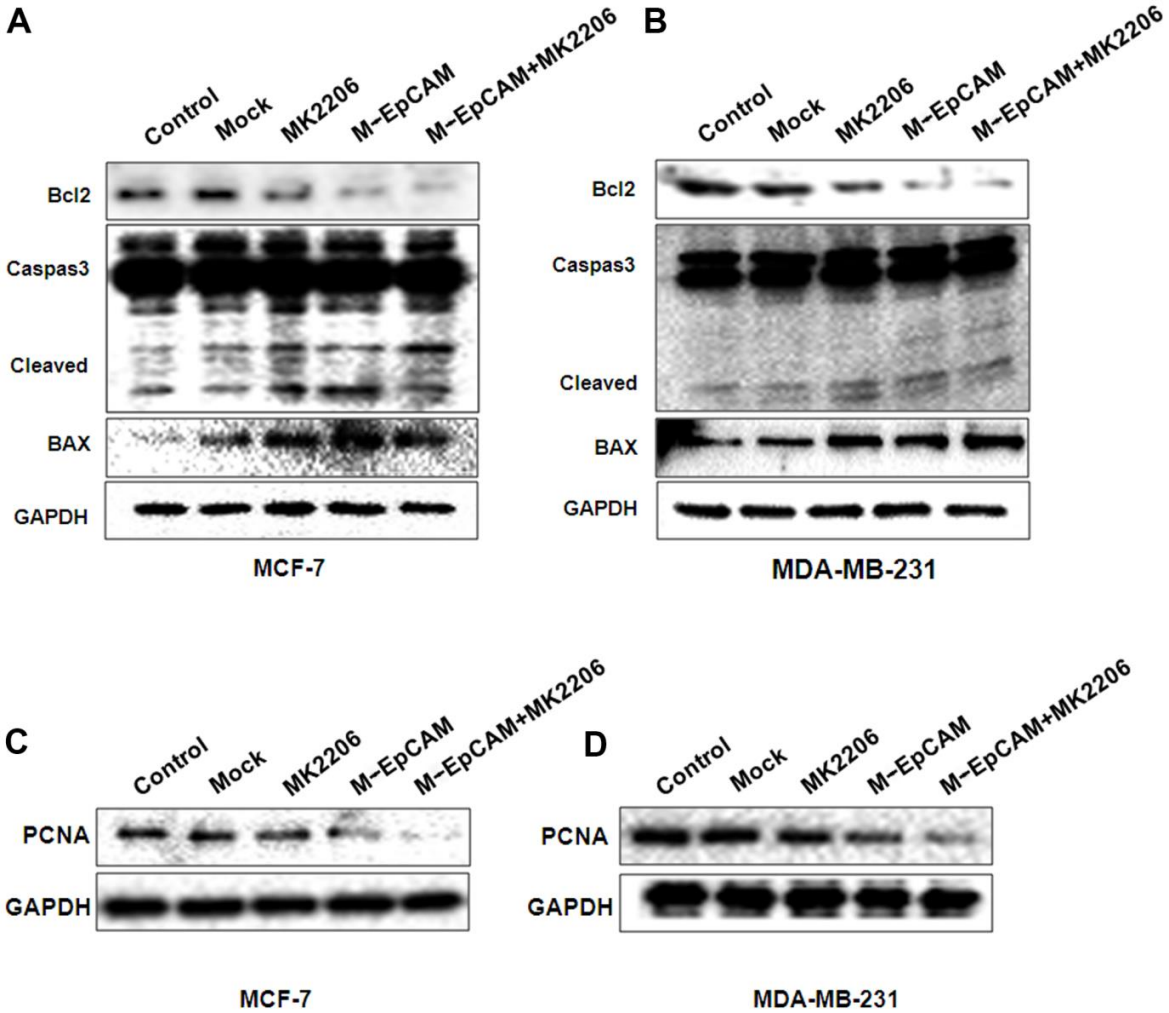
**Effects of EpCAM and autophagy on proliferation and apoptosis via PI3K/AKT/mTOR signaling pathway in MCF-7 and MDA-MB-231 cells.** MCF-7 (**A**, **C**) and MDA-MB-231 (**B**, **D**) cells were treated with M-EpCAM plasmid and MK2206 (1μM) for 48 h. Whole cell lysates were subjected to western blot to detect the expression of Caspase 3, Bcl2, Bax and PCAN.

**Figure 8:**
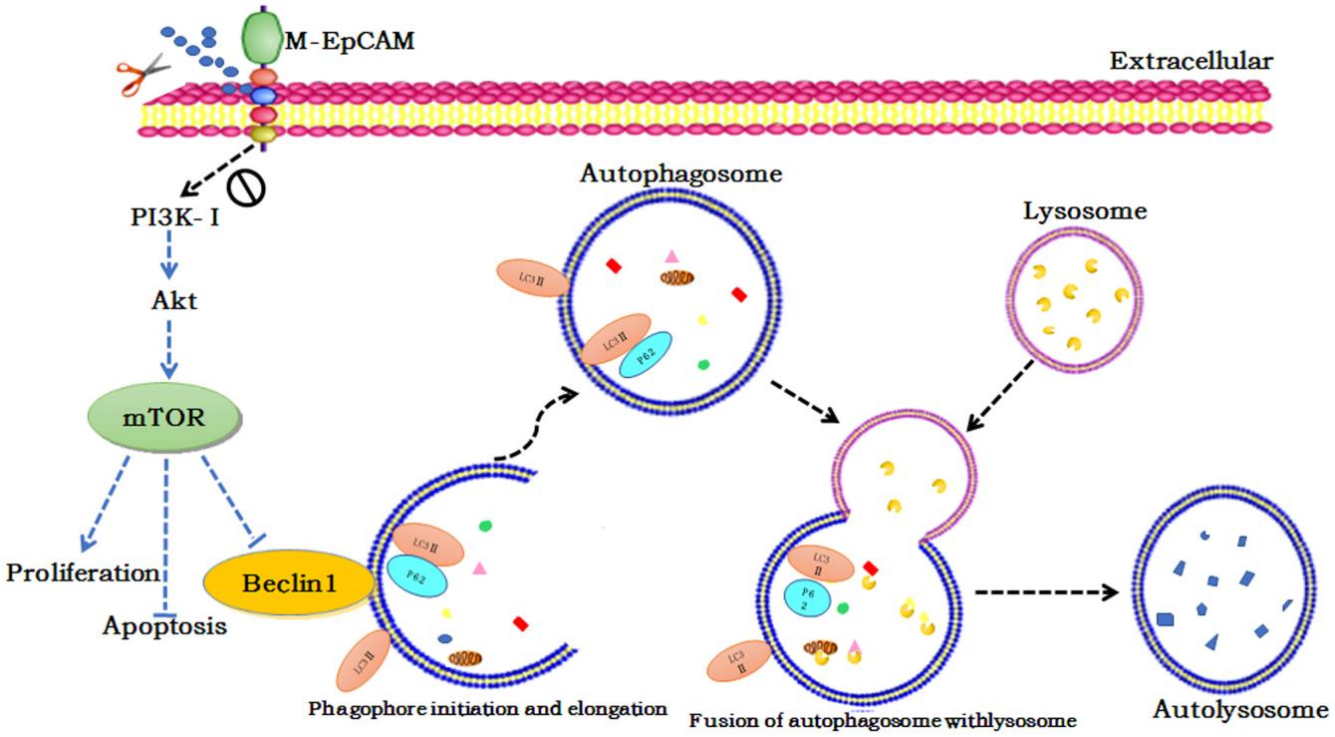
Schematic diagram of the proposed mechanism underlying deglycosylated EpCAM-induced autophagy participated in the proliferation an apoptosis viaI3K/Akt/mTOR signaling in breast cancer cells.

We also investigated the effect of glycosylation modification of EpCAM combined with inhibitor of Akt (MK2206) on apoptosis and proliferation in BC (Figure 7). In the MK2206 treated cells and M-EpCAM treated cells, cleaved-caspase 3 and BAX were increased and Bcl2 was decreased. When combined MK2206 and M-EpCAM, the results showed more effective. As shown in Figure 7C, 7D, PCNA expression in the MK2206 and M-EpCAM treated cells were decreased deeply. These data clearly demonstrated that glycosylated EpCAM plays a critical role in regulating autophagy, apoptosis and proliferation on breast cancer cells. Taken above, these data indicate that deglycosylated EpCAM-induced autophagy participated in the proliferation an apoptosis viaI3K/Akt/mTOR signaling in breast cancer cells (Figure 8).

## DISCUSSION

Autophagy is a catabolic process necessary for development, innate immunity, cellular stress responses, and cell death in physiology and pathology conditions. During this process, long-lived proteins overturn constitutively and macromolecules and organelles are damaged [2, 21, 25]. Autophagy plays an important physiological role to maintain cellular homeostasis [26]. Abnormal activation of autophagy can induce cell death [27]. Whether autophagy promotes cell survival or induces cell death is controversial. Through our study, we illustrated that autophagy and subsequent apoptosis were induced by deglycosylated EpCAM in breast cancer cells. Furthermore, we demonstrated the essential role of PI3K/Akt/mTOR signaling pathway in deglycosylated EpCAM-induced autophagy.

The dysregulation of autophagy has been identified in various diseases including cancer, cardiovascular disease, and autoimmune disease [15]. Till now, many autophagy related proteins and modulators have been found to involve in the autophagy [28]. Autophagy plays a dual role in cell survival and death in both normal tissues and tumors [8]. The interplay between autophagy and cancer cell development and proliferation is complex. Autophagy has been implicated to be closely related to tumorigenesis [29]. Some evidence implicates autophagy as a tumor suppressor, while other evidence suggests that it promotes tumor proliferation. Thus, autophagy plays different roles in cancer biology depending on tumor type and context [9].

Glycosylation is the common protein modification after translation. Abnormal glycosylation influence cancer development and metastasis. [30]. Tumor cells display a wide range of glycosylation alterations, and certain glycans are well-known markers of tumor progression, such as LeY, sLeX [14, 31]. Some data suggested that glycoconjugates influence physiological behavior by regulating autophagy. The mechanics of autophagosome formation was associated with glycoconjugates [32]. Many data have demonstrated that the relationship between the glycosylation and autophagy. Mutations in other crucial autophagy proteins have also been identified and support the idea that autophagy has a tumor suppressor function. For example, Zhu reported that O-GlcNAcase (OGA) inhibitors enhanced autophagy, which aided the brain in struggling with the accumulation of toxic protein species [17]. Li reported that knockdown of HIF-1α regulated autophagy mediated glycosylation in oral squamous cells [33].

Autophagy and apoptosis, two important physiological behaviors, control cell survival and death in response to various stresses [34]. Autophagy is a conserved process to maintain cellular homeostasis, consisting of the degradation of organelles and abnormal proteins. Autophagy can be rapidly induced by hypoxia, and oxidative stress and so on [35]. Apoptosis is a process which show nuclear shrinkage, chromatin condensation and apoptosis body [36]. In some cases, autophagy can inhibit apoptosis for cell survival, while in other cases autophagy may lead to cell death [37, 38]. Therefore, the combination of autophagy and apoptosis affects cell homeostasis. Many studies have shown the interaction between autophagy and apoptosis. This interaction is mainly reflected in the interaction between autophagy protein and apoptotic protein, such as Bcl-2/Beclin-1, Atgs, Caspases, p53, FLIP, and so on [39]. Therefore, it is of great significance to explore the interaction between autophagy proteins and apoptotic proteins. In general, autophagy predates apoptosis in maintaining cell homeostasis. Autophagy may become a guardian of apoptosis through surrounding microenvironment [40]. Existing literature reported that autophagy participated the process of cell apoptosis. For example, autophagy-dependent apoptosis was regulated by inhibition of SGK1 via the mTOR-Foxo3a pathway [41].

We reported that deglycosylated EpCAM induced autophagy and apoptosis in breast cancer cells in this study. Blockade of autophagy with autophagy inhibitor Wortmannin completely inhibited deglycosylated EpCAM-induced autophagy apoptosis in breast cancer cells. Activation of autophagy with autophagy activator Rapamycin got the opposite results. It has been demonstrated that a variety of integral cell signaling pathways are known to regulate autophagy.

The PI3K/Akt/mTOR signaling pathway is the common way to regulate autophagy [42, 43]. Akt is phosphorylated and activated by PI3K. pAkt activates the downstream mTOR and make it phosphorylated, which is followed by down-regulation of phosphorylated p70S6K. The result induces autophagy [6, 13, 42, 44, 45]. To understand the mechanism of deglycosylated EpCAM-mediated autophagy in breast cancer cells, we analyzed the Akt-mTOR signaling pathway. Our study demonstrated that the effect of Akt-mTOR signaling on the regulation of autophagy in breast cancer cells. In addition, we also suggested that deglycosylated EpCAM facilitated cell apoptosis of breast cancer cells via PI3K/Akt signaling pathway.

## CONCLUSIONS

In summary, we reported the autophagy disorder induced by three glycosylation point mutations in EpCAM in breast cancer cells. Deglycosylated EpCAM was found to be a functional marker that was required for AKT/mTOR mediated autophagy regulation in breast cancer cells. Our study further explore EpCAM functions and provides a theoretical basis for the treatment of breast cancer.

## MATERIALS AND METHODS

### Materials

MCF-7 and MDA-MB-231 cells were obtained from ATCC (Manassas, VA). DMEM/F12, fetal bovine serum (FBS), Lipofectamine TM Reagent was purchased from Invitrogen (Paisley, UK). The anti pAkt, Akt, pmTOR, mTOR, GAPDH and horseradish peroxidase (HRP)-conjugated anti-rabbit secondary antibody were obtained from Santa Cruz Biotechnology (Heidelberg, Germany). The anti Beclin 1, P62, ILC3, Caspase 3, Bcl2 and Bax were purchased from Proteintech (Wuhan, Hubei, China). Enhanced chemiluminescence (ECL) assay kit was purchased from Amersham (Louisville, Co).

### Plasmid and RNAi sequence

EpCAM overexpression plasmid (pCMV-SPORT 6-EpCAM) was purchased from the Proteintech Group, Inc. (Wuhan, Hubei, China). SiRNA for EpCAM is as follows: EpCAM-1: 5′-UGCUCUGAGCGAGUGAGAATT-3′; EpCAM-2: 5′-UUCUCACUGCUCAGAGCATT-3′. N-glycosylation mutant was made by replacing asparagine with glutamine in all the three N-glycosylation sites of EpCAM by TAKALA Company (Dalian, Liaoning, China). The mutation plasmid was named M-EpCAM plasmid.

### Cell culture

Breast cancer cells (MCF-7 and MDA-MB-231) were cultured in medium DMEM/F12 plus 10% calf serum in a 5% CO_2_ humidified atmosphere at 37° C.

### Transfection

Transfection of cells with EpCAM overexpression plasmid, si-EpCAM sequence, M-EpCAM plasmid and pGFP-LC3 plasmid were performed using LipofectamineTM Reagent according to the manufacturer’s instruction.

### Western blot

Cell extracts were prepared using with RIPA lysis buffer. After determination of concentration of protein, cell lysates were separated by 10% SDS-PAGE min-gel and transferred to a nitrocellulose membrane. Subsequently, the membrane was blocked using 5% non-fat milk for 2 h and then incubated with the primary antibodies overnight. After probed with horseradish peroxidase-conjugated secondary antibodies, immunoreactive proteins were visualized with ECL detection system.

### Cell proliferation assay

Cells (1×10^4^ cells per well) were seeded in a 96-well plate. Cell viability was performed using an CCK8 assay, according to the manufacturer's instructions. The absorbance of OD_450nm_ was detected with a microplate reader.

Each sample was evaluated for three times for analysis.

### Electron microscopy

Cells were treated with 2.5% glutaraldehyde in 0.1 M sodium cacodylate buffer, pH 7.4 for 30 minutes at room temperature and post-fixed with 1% osmium tetroxide in 0.1 M sodium cacodylate buffer, pH 7.4 for 1 hour, contrasted with 1% tannic acid in 0.05 M sodium cacodylate, followed by dehydration through graded alcohols and acetone and then embedded in EMbed 812. After using an UltraCut E ultramicrotome to cut into Ultrathin sections, samples were double-stained with 0.3% lead citrate and examined under a JEOL 1200EX electron microscope. Micrographs were taken at 60,000 or 200,000 magnifications.

### Statistical analysis

The numerical data are expressed as means ± SD. Unpaired Student’s t-tests were used to compare the means of two groups. *P* value less than 0.05 was considered significant.
